# Synchrony perception of audiovisual speech is a reliable, yet individual construct

**DOI:** 10.1038/s41598-025-00243-8

**Published:** 2025-05-07

**Authors:** Liesbeth Gijbels, Kaylah Lalonde, Yi Shen, Mark T. Wallace, Adrian KC Lee

**Affiliations:** 1 Department of Speech & Hearing Sciences, University of Washington, Seattle, Box 357988, 98195-7988 WA USA; 2 Institute for Learning & Brain Sciences, University of Washington, Seattle, WA USA; 3 Center for Hearing Research, Boys Town National Research Hospital, Omaha, NE USA; 4 Department of Hearing and Speech Sciences, Vanderbilt University Medical Center, Nashville, TN USA; 5 Vanderbilt Brain Institute, Nashville, TN USA; 6 Department of Psychology, Vanderbilt University, Nashville, TN USA

**Keywords:** Temporal binding window, Temporal sensitivity, Audiovisual, Speech perception, Reliability, Linguistic, Auditory system, Sensory processing, Visual system, Human behaviour

## Abstract

Audiovisual (AV) (a)synchrony perception, measured by a simultaneity judgment task, provides a proxy measure for the temporal binding window (TBW), the interval of time within which participants perceive individual auditory and visual events as synchronous. The TBW is a sensitive measure showing group level differences across the lifespan. However, the significance of these findings hinges on whether AV synchrony perception is characteristic to an individual and whether it is reliable across sessions. As there is little evidence to this latter assumption, this work aimed to establish test-retest reliability of the TBW. Eighteen participants completed a simultaneity judgment task, twice, on different days, where they judged whether or not the audio and video of word-length speech stimuli were presented at the same or different times. Results showed effects of task familiarity indicated by significantly faster response times observed at the second timepoint. The slope and amplitude asymptote of the TBW also increased, however no change in TBW suggests no change in sensitivity to AV (a)synchrony. The combination of high within-subject correlations (*R* = 0.71) and substantial intersubject variability provide strong support for the TBW as a robust measure of AV temporal perception, and suggest a conserved mechanistic underpinning within individuals.

**Supplementary Information:**

The online version contains supplementary material available at 10.1038/s41598-025-00243-8.

## Introduction

Incorporating visual information from the talker’s face and mouth enhances auditory speech perception, especially in challenging listening environments^1–4^. This complex, yet pivotal process of integrating audiovisual (AV) information to improve our speech understanding relies on solving the causal inference problem; more specifically, the decision if and how to integrate information from the auditory and visual streams^5^. This process is based on coherence of both information streams^6^, with temporal information being arguably one of the most important cues in this process^7,8^.

A variety of temporal cues play an informative role in AV speech identification (e.g., temporal envelope tracking^9^; natural rhythmic head motion^10^). Furthermore, the temporal structure of AV signals plays a key role in the likelihood of object formation, as evidenced by studies examining single neurons^11^, neural populations^12,13^, behavior^14–16^, as well as in observations among neurodiverse groups (such as those on the autism spectrum ^17^).

In naturalistic settings, temporal information from the same object or event is strongly correlated in the auditory and visual modalities. Nevertheless, and especially for AV speech stimuli, there is often no absolute synchrony. Visual information precedes auditory information in conversational contexts as the timing of the mouth movements precedes the onset of the voice by tens to a few hundred milliseconds^18,19^, and visual information reaches the listener sooner than auditory information due to differences in the speed of propagation of electromagnetic versus acoustical waves.

Fortunately, it is the perception of synchrony, and not absolute synchrony, that is crucial for integrating AV speech. Our perceptual system can accommodate a range of signal asynchronies (viz., up to a few hundred milliseconds) and perceive them as simultaneous^20^. Following this, the Temporal Binding Window (TBW) is operationally defined as the range of Stimulus Onset Asynchronies (SOAs) within which stimuli from different sensory modalities are perceived as simultaneous^21,22^.

When assessing the TBW, participants are exposed to stimuli in two different modalities that can be generated from the same source (e.g., a video of a hammer hitting with the sound of a hammer, or a speech signal with a matching video of the talker). Stimuli from these two modalities are presented synchronously (SOA = 0 ms), or asynchronously (often up to SOA = 500 ms), and in a simultaneity judgment (SJ) task participants must judge the synchrony/asynchrony of these presented stimuli. The asynchrony can be either audio-leading or visual-leading, and the response distributions, representing the percentages of synchronously perceived responses, are generally fit to two separate psychometric functions, one for audio-leading and one for visual-leading responses (see^23^) for details on different test paradigms). From these psychometric functions, two key measures are retrieved: the SOA corresponding to the highest probability of reports of simultaneity (or Point of Subjective Simultaneity; PSS) and the width of the TBW. The TBW is often defined as the width of the conjoined distributions at which simultaneity responses are either 50%^22,23 ^or 70%^23,24^.

The TBW width measure has been successfully employed in studies showing group level differences in AV temporal acuity across the lifespan^25^, as well as in studies that have highlighted a connection between the TBW measure and specific neurodevelopmental disorders (for a review^26^). Furthermore, a significant body of literature delves into the perception of AV (a)synchrony as an indicator of the magnitude of AV integration, as there is a negative correlation between AV (a)synchrony perception (i.e., the TBW width) and auditory and AV speech perception performance^27,28^, AV speech perception benefits^29^, and the perception of AV illusions^29^. This implies that individuals vary in their AV (a)synchrony perception based on how much they employ AV cues (in AV benefits and AV illusions).

The significance of this body of work hinges on two critical factors: (1) the test-retest reliability of the TBW measures (i.e., within-subject consistency) and (2) whether there is significant variability in temporal synchrony perception across individuals. As this second point has been observed, even within a rather homogenous group of younger adults with typical hearing^28^, one could argue that if intersubject variability is found to be significantly greater than intrasubject variability, it would provide support for the TBW paradigm as an assessment tool for AV temporal acuity, and by extension, of the magnitude of AV integration at the individual level.

Interestingly, to the best of our knowledge, there are very limited reports of the test-retest reliability of the TBW on a group or individual level (preprint^30^). Hence, our current study seeks to examine the stability of the TBW within individuals across multiple sessions as well as gauge the degree of variability in this measure across a population.

To do so, a group of 18 younger adults (mean age = 28.5 years old, min = 21, max = 40) completed a remote, but offline, simultaneity judgment task (see Methods for details) at two separate test intervals (within a month from each other), in the comfort of the participants’ homes on their personal computers. Participants were presented with synchronous or asynchronous AV speech stimuli (0–500 ms visual-leading), and had to decide whether the audio and video were presented at the same or different times.

We developed a remote measurement method for TBW assessment, yielding shapes and sizes consistent with previous in-person measures^15,22,31^, while acknowledging that the intersubject variability reported here included the additional variability associated with remote testing. To ensure quality of responses of the participants, we defined and excluded outliers (see supplemental materials, Figure S1) before analyzing inter- and intra-subject reliability of the TBW, defined by the width, slope and asymptote amplitude of the psychometric functions. One participant was excluded based on outlier criteria (i.e., residual scores, significantly deviating response times, or response scores), leaving data from 17 participants for further analysis.

All speech stimuli were of word length (1-, 2-, or 3-syllables) and were presented in background noise (female 4-talker-babble). The linguistic characteristics of the stimuli varied, as we used words, nonsense words (which have the same amount of phoneme-viseme information available and the same duration as words but no meaning), and reversed words (i.e., words or nonsense words played backwards; thus same duration but no meaning and no phoneme-viseme connections between the auditory and visual signal). However, as the differences in temporal synchrony judgment caused by differences in linguistic information of speech stimuli are beyond the scope of this manuscript, we will use the different stimuli types as three separate indicators of inter- and intra-subject reliability of the TBW. We will refer to them from here on out as Type A (i.e., word), Type B (i.e., nonsense word), and Type C (i.e., reversed word) speech stimuli, and assess inter- and intra-subject reliability for all types separately, allowing us to compare within and between different stimuli types.

## Results

### TBW shape

#### Group level analysis

To investigate whether sensitivity to AV (a)synchrony can reliably be represented by the TBW, we first looked at the replicability of our TBW shape at the two different test intervals on a group level. The TBW shape is represented by a psychometric function defined by the width, the slope and the asymptote amplitude for synchronous or visual-leading asynchronous AV stimuli. We did not expect the width of the TBW, defined at the 50%-synchrony perception point, to differ at the two time intervals, based on results from a study of Powers and colleagues^32^, who investigated whether sensitivity to AV (a)synchrony could be improved by training; and thus whether the TBW width could be changed by training. They used a similar task (i.e., 1I-2 AFC-SJ; however with flashes and beeps) and found no significant differences in sensitivity to temporal (a)synchrony at the baseline testing and the first day of training (i.e., pre training) on a group level.

**Fig. 1:**
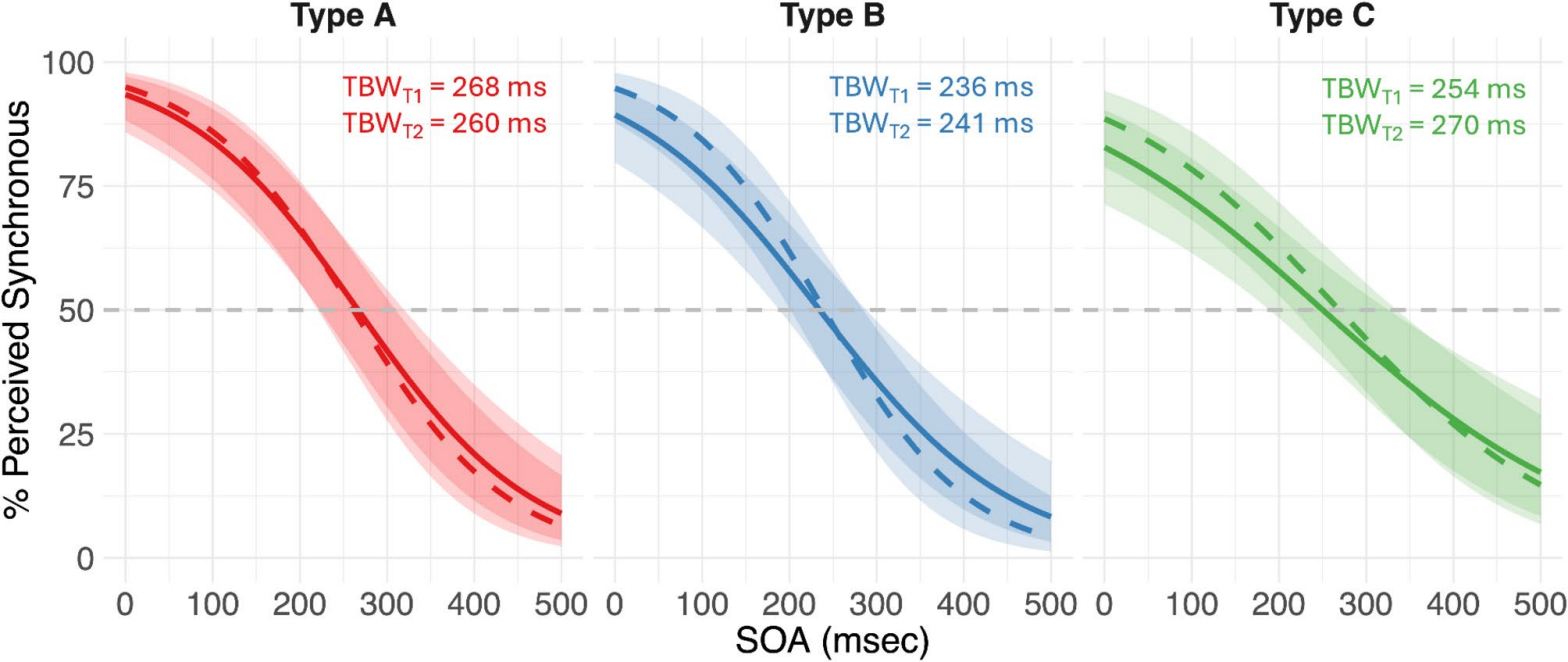
The Temporal Binding Window of the 3 different stimulus types (**A**, **B**, **C**) for the two different test moments.

We used a linear mixed effects model (Model 1a, see Methods) to analyze the influence of test interval and stimulus type (and the interaction of those two) on the sensitivity to AV (a)synchrony detection, quantified by the width of the TBW. The results showed no main effect of test moment on the 50% TBW width (F_1,80_ = 0.15, *p* = 0.6945), showing that the TBW did not significantly differ between T1 and T2 (Fig. 1). There was a main effect of stimulus type (F_2,80_ = 7.71, *p* = 0.0009), yet no interaction effect (F_2,80_ = 1.75, *p* = 0.1806). These results thus show that, at least on a group level, our temporal (a)synchrony perception measured by the TBW width at the 50%-synchrony perception point did not significantly change at different test moments.

As the main effect of test moment on the 50% TBW width is our primary measure of interest, and given that the interpretation of a null effect in a linear model can be arbitrary, we used the Bayes Factor to quantify the evidence in favor of the null hypothesis (Bayes Factor < 1). Our results yielded a Bayes Factor of 0.18 ± 0.07%, indicating that the data are approximately 5.56 times more likely to support the null hypothesis (H₀) than the alternative hypothesis (H₁). This provides moderate evidence in favor of the null effect, suggesting that there is no significant difference in TBW width between T_1_ and T_2_. Furthermore, we calculated the Bayes Factor for different sample sizes (by randomly selecting participants (N: 1–17) from our data set) and repeated this 10 times. These analysis show stable performance when *N* > 13, suggesting our rather moderate sample size (*N* = 17) is sufficient to show a null effect between both test moments (See supplementary figure S2).

Test moment 1 (T1) = full line, test moment 2 (T2) = dashed line. Percentage perceived synchronous is plotted in function of SOA, ranging from 0 to 500 msec. The psychometric functions are calculated based on a general linear model with a binomial distribution (logit link function) pooled across participants, and the 95% confidence interval is plotted as a shaded band around the psychometric function. The cross-section of the psychometric function and the horizontal gray dashed line represent the 50% point of perceived synchrony. The TBW width is obtained by extrapolating the cross-section of this horizontal line with the sigmoid function to x-axis, resulting in the SOA that corresponds to 50% synchrony perception (right top corner).

The slope of the psychometric function (Model 1b, see Methods), did show a main effect of test moment (F_1,80_ = 30.09, *p* < 0.0001), and stimulus type (F_2,80_ = 44.79, *p* < 0.0001), and a marginally significant interaction effect (F_2,80_ = 3.31, *p* = 0.042). Thus, when participants complete an AV (a)synchrony decision task for a second time, at least on a group level, the TBW shape becomes significantly steeper, suggesting that the sensitivity to (a)synchrony changes more rapidly with increasing SOA when the task is repeated. The main effect of slope on stimulus type then shows that the change of sensitivity to temporal (a)synchrony by SOA within the same cohort of participants is different, based on the stimuli used.

The asymptote amplitude of the psychometric function (Model 1c, see Methods), i.e., the difference between the maximum and the minimum synchrony perception score also showed a main effect for both test moment (F_1,80_ = 10.71, *p* = 0.0016), and stimulus type (F_2,80_ =25.91, *p* < 0.0001), and no interaction effect (F_2,80_ = 1.12, *p* = 0.332). All three stimulus types showed a more pronounced psychometric function at T2. Thus, a larger asymptote amplitude, as reported in the second test moment, compared to the first, suggests that participants show increased consistency about the AV (a)synchrony decision.

In sum, we found no significant change in TBW width measures between T1 and T2 for the 50% synchrony perception point, however the slope and asymptote amplitude significantly changed, showing as a larger (closer to 0% and 100% synchrony perception) and steeper sigmoid-like psychometric function, at T2. These results indicate that the point of perceived AV synchrony (i.e., TBW width at 50% synchrony perception) does not change significantly by repeating the task, yet the change in slope and asymptote amplitude likely indicates increased certainty about the AV (a)synchrony decision.

#### Individual level analysis

##### Test-retest reliability of the TBW - intrasubject variability

**Fig. 2:**
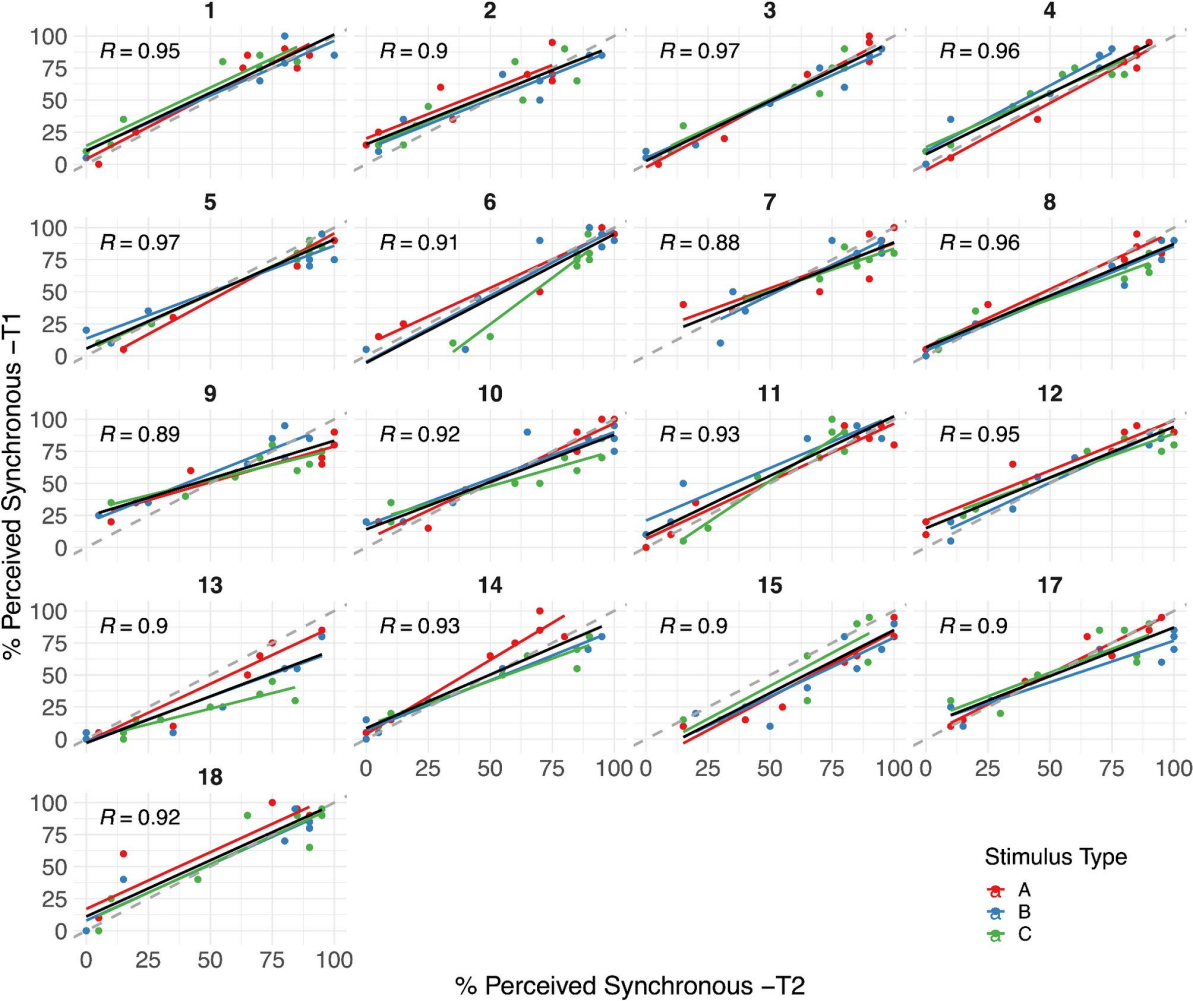
Correlational plots of temporal synchrony judgments per individual participant, per stimulus type, and stimulus types combined.

**Fig. 3:**
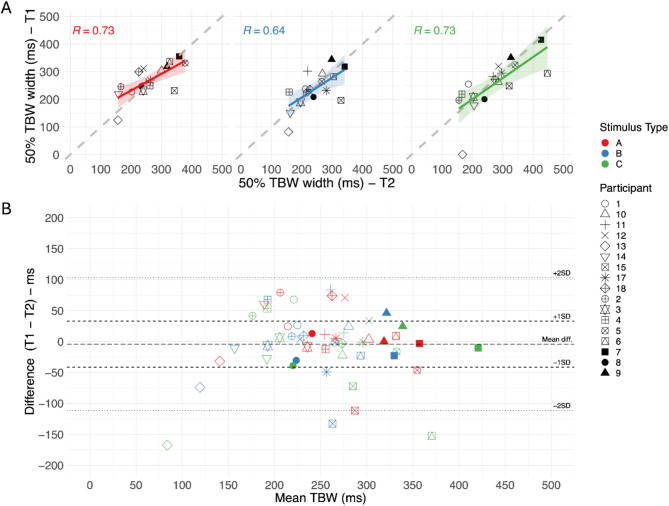
TBW width comparison at two timepoints.

Test-retest reliability at an individual level was interpreted by using Pearson correlation coefficients. We measured the correlation between T1 and T2 (per stimulus type level) for each participant, based on the actual percentage perceived synchrony score (Fig. 2) and the TBW width (50% synchrony perception point; Fig. 3). A strong correlation between the TBW width at both test moments would indicate a satisfactory test-retest reliability.

Due to the sigmoid-like nature of the TBW, the actual percentage of perceived synchrony scores tended to cluster predominantly in the lower-left and upper-right regions (Fig. 2), reflecting a majority of responses classified as either synchronous or asynchronous. If strongly correlated, the scores should fall on the diagonal (dashed gray line), indicating that performance at both test moments was identical. For most of the participants the correlation plot shows a nice approximation of the diagonal for each stimulus type (R_Type A_ = 0.93, t = 30.21, *p* < 0.0001; R_Type B_ = 0.92, t = 27.03, *p* < 0.0001; R_Type C_ = 0.88, t = 21.38,*p* < 0.0001). Averaged across stimulus types, we observed a very strong Pearson correlation coefficient of *R* = 0.91 (t = 45.06; *p* < 0.0001), indicating that participants were consistent in their synchrony decision for each SOA at both test moments.

The Pearson correlation coefficients for the TBW width between the two test moments (Fig. 3A), also showed an overall strong correlation (*R* = 0.71, t = 6.97, *p* < 0.0001), translated to each specific stimulus type (R_Type A_ = 0.73, t = 4.14, *p* = 0.0009; R_Type B_ = 0.64, t = 3.25, *p* = 0.0054; R_Type C_ = 0.73, t = 4.17, *p* = 0.0008). These correlation coefficients provide us a value of consistency, which reflects the extent to which the participants are ranked in the same pattern at the two timepoints. We further assessed agreement of the TBW between test moments by calculating the intraclass correlation coefficient (ICC), using a single-rater mixed effects model, and obtained an ICC of 0.82 [0.68; 0.90]. The model estimates variance components using restricted maximum likelihood, allowing representation of accurate ICC estimates, even with sample sizes as small as *N*= 10^56^.

By constructing the Bland-Altman plot (Fig. 3B) we observe that the difference between the TBW between the two timepoints, on average is just − 4 ms, and that for 68% (1 sd) of our data points the difference falls between − 41 ms and + 33 ms of each other. More details can be found in supplemental materials table TS2.

Both axes plot the percentage synchrony perception. The y-axis represents test moment 1 (T1), and the x-axis test moment 2 (T2). The dashed gray diagonal line represents the ideal scenario where performance on both test moments is identical. The stimulus types are presented by different colors (red: Type A, blue: Type B, green: Type C), and the black line is not split up by stimulus type. Each individual dot is the percentage simultaneity perception for 1 SOA – as noted earlier, P16 was excluded from the analysis. The Pearson correlation coefficient, averaged across the three stimulus types, is plotted in black in the top left corner for each participant.

Each individual shape is a participant. The colors represent the different stimulus types (A, B,C). Top: Correlational plots of TBW width per stimulus type. Both axes show the TBW (in ms, based on the 50% synchrony perception point of the sigmoid function). The y-axis represents test moment 1 (T1), and the x-axis test moment 2 (T2). The dashed gray diagonal line represents the ideal scenario where performance on both test moments is identical. The colored line is the regression line per stimulus type, and the shaded area is the 95% confidence interval. Pearson correlation coefficients and p-values are plotted in the left top corner. Bottom: Bland-Altman plot, a scatter plot representing the differences between the TBW width at the two timepoints (y-axis), plotted against the average TBW across two timepoints (x-axis), for the different participants and different stimulus types. The plot shows the mean difference (long dashed line), 1 sd (dashed lines) and 2 sd (dotted lines) from the mean difference.

In sum, the strong linear correlation between the two test moments for both percentage synchrony perception at each SOA (*R* = 0.91, t = 45.06, 0.0001), and the estimated TBW width (*R* = 0.71, t = 6.97, *p* < 0.0001), shows good intra-subject test-retest reliability (within participants) of the TBW measure, tested at different test intervals. The ICC score of 0.82 [0.68; 0.90] is a further indication of good reliability between both test moments, and the highly correlated TBW width scores between the different stimulus types (Table 1) further establish the reliability of the TBW measure within an individual.

**Table 1:** Pearson correlation coefficients of TBW width between stimulus types.

Stimulus Type	*R*	95% CI	t-value	*p*-value
Type A vs. Type B	0.82	[0.66–0.91]	8.09	< 0.001
Type A vs. Type C	0.85	[0.71–0.92]	9.05	< 0.001
Type B vs. Type C	0.87	[0.75–0.93]	9.93	< 0.001

The table represents the Pearson correlation coefficient (R), with 95% confidence interval, t- and p-value between the 3 stimulus types. This is combined for test moments 1 and 2.

##### Inter-rater reliability of the TBW - intersubject variability

When intrasubject variability (within participants; as described in the section above) is smaller than intersubject variability (the range of TBW width across participants), even in a relatively homogeneous group (neurotypical young adults, as tested here), strong evidence is provided for the reliability of the TBW measure.

From Fig. 3 (top), we observed that individuals with narrower/wider TBWs in test moment 1, also have narrower/wider TBWs in test moment 2 and that individuals that have narrower/wider TBWs for one stimulus type, also have narrower/wider TBWs for other stimulus types (Table 1). For example, in the current cohort of participants, participant 14 has one of the narrowest TBW widths for all test moments and all stimulus types, whereas participant 7 has one of the widest.

To quantify this intersubject variability, the interquartile range (IQR: Q3-Q1) was selected. For Type A stimuli the TBW width ranges from 125 ms to 378 ms, with an IQR of 86 ms. The IQR was 73 ms for Type B [range: 82.13 ms − 344.39 ms], and 110 ms for Type C stimuli [range: 0 ms − 446.65 ms]. Comparing the variance of the TBW width within and between participants, by using a likelihood ratio test, a significant effect (χ² = 10.44; *p* = 0.001) shows that the variance between participants (S^2^ = 3503.18, sd = 59.19) is about five times as large as the variance within participants between test moments (S^2^ = 685.64, sd = 26.18).

The results thus show that when individuals repeat an AV (a)synchrony perception task, they are more consistent on the second test. Nonetheless, the AV (a)synchrony measure represented by the TBW width shows large intersubject, but small intrasubject variability, suggesting its utility as a measure to study individual differences in AV temporal acuity.

### Response times

#### Group level analysis

Participants received no instruction on the timing of completion of the tasks, but were asked to complete the task to the best of their abilities. We did, however, collect the response time of each participant for each trial, at T1 and T2. Response times here are defined as the time from the start of the stimulus presentation to the key press entered by the participant. The participant could complete a trial from the start of the stimulus presentation, and could, but did not have to, wait for the entire video to play. A change in response times across test moments could inform us about time needed for decision making based on familiarity with the task.

**Fig. 4:**
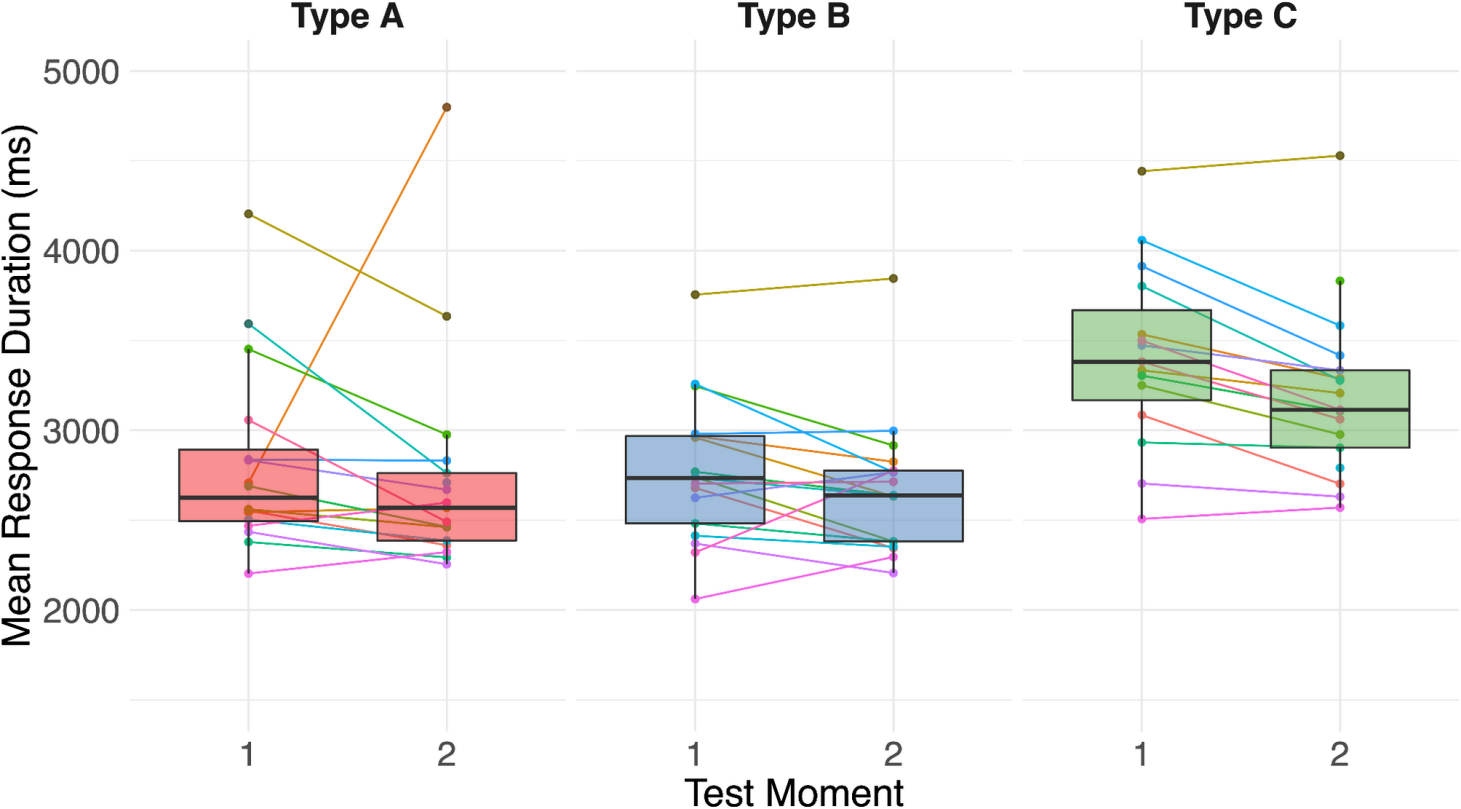
The Mean Response Duration of the 3 different stimulus types (**A**, **B**, **C**) for the two different test moments.

We used a generalized additive mixed model (Model 2, see Methods) to investigate the effects of stimulus type, test interval, and the interaction between these two on response times. The model showed a main effect of both test moment and stimulus type on response time. No significant interaction effect was reported. Responses were significantly faster at T2 (_t1_ = 3134 ms; _t2_ = 2870 ms, t = −3.39, *p* = 0.0007), showing that, on average, participants were able to complete the trials faster in the second session, regardless of the stimulus type (Fig. 4). Thus, when participants were more familiar with the task, a decision regarding (a)synchrony of the AV stimuli was made faster. In further investigating the main effect of stimulus type, we note that response times for Type A and Type B stimuli were not significantly different; however, responses for both of these stimulus types were significantly faster (i.e., _Type C_ = 3442 ms; _Type A_ = _Type C_ −599 ms, _Type B_ = _Type C_ −720 ms) than response times for the least familiar (i.e., reversed word) stimulus (Type C; t_A−C_ = −4.63 (*p* < 0.0001), t_B−C_ = −5.83 (*p* < 0.0001)).

Mean response time in ms by test moment (1 (left) or 2 (right)). Boxplots show group level results for stimulus Type A (red), B (blue), and C (green). First, second and third quartiles are represented by black horizontal lines. Individual participants are represented by colored dots, and the change of each participant from test moment 1 to 2 is represented by a line.

#### Individual level analysis

Response times of individuals were mostly conserved across test intervals (i.e., the slower individuals in test moment 1 were also relatively slower in test moment 2), albeit the absolute response time in test moment 2 was slightly faster (Fig. 4). This response time pattern was conserved across the three different stimulus types.

## Discussion

Audiovisual (a)synchrony perception, measured by the TBW width, has been regularly described as an indicator of individual differences in the temporal integration of AV signals. The TBW has been reported for both AV non-speech and speech stimuli (i.e.^23^ for comparison of stimuli and tasks), from children to older adults^25^, and in neurotypical and neurodiverse populations (^21 ^for a review). The validity of the TBW as an indicator for individual differences in sensitivity to AV (a)synchrony requires the task to show small intrasubject variability in comparison to the intersubject variability. Despite this, we have limited information about the test-retest reliability of the TBW within and between individuals. It was suggested that the PSS – the point where the highest synchrony perception is reached in an SJ task – is variable both between and within individuals^33^. However, this might have been more of a task effect than a test-retest reliability effect^34,35^. An in-lab versus online comparison of a different task^30^, the AV flash-beep illusion, did show a reliable PSS within the task (*R* = 0.75), and a highly reliable TBWs within individuals at two test moments (*R*= 0.93). Nonetheless, care in interpretation of the TBW as it relates to illusions^30 ^is warranted as these might be driven more by the nature of the task rather than integration that would occur for naturalistic speech signals^36^.

The findings of this earlier work^30 ^comparing in-lab and online TBW measures address concerns about using small temporal differences as a measure of interest in a remote format. While we recognize that hardware differences among participants could have inflated the intersubject test-retest reliability, we took several steps to mitigate this issue. Lab.js, the platform we used, has demonstrated very low inter-trial variability (< 9 ms) for auditory, visual, and audiovisual stimuli^45^. To further reduce potential delays, we conducted the task offline. Additionally, we designed the task to be incompatible with mobile devices (e.g., phones or tablets) and ensured to only run reliably in designated browsers (i.e.,Google Chrome or Firefox, but not others e.g., Safari). These measures were implemented to minimize hardware-related variability across participants.

The current work compared AV speech stimuli (of different complexity: words, nonsense words, and reversely played words and nonsense words) at two different test intervals (on different days), to interpret the test-retest reliability of the TBW, and more specifically, the variability of the TBW width (i.e., 50% synchrony perception point) on an individual level as an indicator for AV (a)synchrony perception.

On a group level, the slope and asymptote of the TBW changed when the task was repeated. The increased consistency in responses, as represented by a steeper slope and a larger asymptote amplitude in test moment 2 for all stimuli, suggests that task familiarity has an effect on AV (a)synchrony decisions. This is not surprising as we also noted an improved response time at T2 vs. T1. Nonetheless, the increased consistency in AV (a)synchrony reports did not affect the TBW width.

Nevertheless, this does not mean the TBW cannot be changed. There is evidence that the TBW width can be altered as (a)synchrony perception training narrows the TBW^32,37,38^even with only exposure to unisensory stimuli^39^. However, simple passive repetition, as in our task, is not sufficient to narrow the TBW. Powers and colleagues^32^ indeed showed no difference in visual-leading TBW width between their baseline condition and pretest training on day 1, or between any of the test moments of their passive exposure experiment. If anything, there was a trend for shifting the full TBW (auditory- & visual-leading) to the right, indicating a shift toward more visual-leading stimuli. Once more, this raises doubts about relying on the PSS as a measure for the integration of AV stimuli, yet advocates for the TBW width as a consistent measure, at least on a group level.

At an individual level we reported strong within-subject correlations (*R*= 0.71), and high intersubject variability (IQR: 73–110 ms) of the TBW width, confirming the value of the TBW width measure to study individual differences in AV (a)synchrony perception. High intersubject variability of the TBW width is consistent with earlier findings^22,28^. Strong within-subject correlations so far have only been reported between different stimulus types^23^, and in different assessment formats (see preprint^30^). Similar to the findings of Stevenson and Wallace^23^, this work reported very strong correlations (*R* = 0.82–0.87) between all three stimulus types, and added strong within-subject correlations (*R*= 0.71) and good agreement scores (ICC = 0.82) between different test moments of the same task with the same stimuli. These findings support the conclusion^23^ that *“as the construct of the TBW shows a remarkable reliability within subjects*,* it suggests that a common underlying neural architecture for the temporal binding of multisensory stimuli most likely originates from the same event.”*

Thus, the current work shows (1) that differences in TBW width by stimulus type are stable across test moments, however the amplitude of the sigmoid can improve when the task is repeated, (2) that ratings of synchrony perception (*R* = 0.93) and the TBW width (*R* = 0.73) are reliable on an individual level, and (3) that the TBW width measure can be used to study individual differences showing from high intersubject and low intrasubject variability.

The results of this study are of key importance as our experiences are continually providing us with AV information at different temporal offsets. The fact that the TBW, a measure of AV temporal acuity, is stable within an individual over multiple testing sessions suggests a stable neuronal circuit mechanism that supports temporal binding. Furthermore, the fact that the TBW varies widely *across* individuals points toward genetic and/or experiential factors that set the window size for a given individual. The role of experience is further supported by prior work showing that the TBW is malleable when individuals are trained in a perceptual plasticity-based protocol. Overall, these findings point to the importance of future developmental studies focused on defining the maturation of audiovisual temporal acuity, as well as on further work in clinical populations to better understand alterations in multisensory temporal abilities. Collectively, we believe that this construct is critical in the formation of higher-order audiovisual percepts, such as speech understanding in both quiet and noise.

## Methods

### Participants

The inclusion criteria to participate in these experiments were (1) being a younger adult (age 18–40-years-old), (2) living in the U.S. (for the purpose of participant payment processing), (3) being a self-identified native English speaker, (4) having self-reported normal hearing thresholds, and (5) having self-reported normal or corrected to normal vision.

Participants were screened for these criteria based on self-report questionnaires. To further verify self-reported normal hearing thresholds, a short sentence recognition task in quiet was administered at the start of the first experiment. During the sentence recognition task, on each trial the participant was presented with an auditory stimulus and had to type the sentence in a text box. No feedback was provided. Only participants who obtained a perfect score on this sentence recognition task in quiet were qualified to proceed with the rest of the experiment.

Of the participants who completed the task at the first timepoint, 18 participants were willing to repeat this task at a second time point within the required timeframe. All participants who completed the TBW measure at two timepoints are presented in Table 2. Everyone completed the second session on a different day, within a month of the first session. The average age was 28.5 y.o, (Q2 = 26.5, sd = 6.02, min = 21, max = 40), 4 participants identified as male, 14 as female.

**Table 2:** Demographic information of participants.

Participant	Computer audio intensity (%)	Timezone (Standard time)	Age	Gender	Race	Hispanic, Latino, or Spanish origin?
1	60	Pacific	26	F	White	no
2	75	Pacific	25	F	Other Asian	no
3	75	Pacific	33	F	White	no
4	74	Central	27	F	White	no
5	100	Eastern	21	M	White	no
6	74	Central	24	M	White	no
7	67	Pacific	34	F	White	no
8	75	Pacific	23	F	Chinese	no
9	100	Pacific	23	M	Filipino	no
10	75	Central	38	F	White	no
11	34	Eastern	34	M	White	no
12	94	Eastern	34	F	White	no
13	82	Central	40	F	White	no
14	50	Eastern	31	F	White	no
15	75	Pacific	32	F	White	yes
16	70	Pacific	21	F	Black or African American	no
17	75	Central	24	F	White	no
18	75	Central	23	F	White	no

Participant number, computer audio intensity setting indicated by participant at test moment 1, timezone as reported by participant’s computer. Self-reported age (in years), gender, race and hispanic origin.

### Recruitment and consent

Participants were recruited at the University of Washington or from other oral or online advertising. All participants provided remote informed consent for the task under a protocol that was approved by the University of Washington’s Institutional Review Board, and all methods were performed in accordance with the relevant guidelines and regulations. All participants completed these tasks remotely/online. Participants were financially compensated at both timepoints.

### Experimental protocol

The participants completed the TBW task twice, at two different timepoints. At both times, the task was completed remotely in the comfort of the participants’ homes on their personal computers. Instructions were provided via email. The task was designed in a free online study builder, Lab.js^40^. Similar remote paradigms have successfully yielded consistent and repeatable results, even for children^41–44^.

Participants were asked to complete the TBW task in front of a computer, using a comfortable distance to the screen, with built-in speakers (no headphones), and in a “a relatively quiet environment without excessive background noise.” The task was completed offline via one of two browsers: a Google Chrome or Firefox browser. At the beginning of Task 1, participants were asked to set the speakers to a comfortable loudness level, based on a repeating English speech fragment with the same RMS amplitude as the test stimuli in the experiment^41–44^. They were requested not to change the volume setting of their computer for the entirety of the experiment and had to provide an estimate of this setting (0–100%), so the same intensity was used for both time points (Table 2).

A folder containing the experimental task was downloaded to their personal computers to run an index .html file. At the end of the experiment a .csv file was generated by the experiment builder, which had to be emailed back to the researcher^41–44^. By executing the experiment remotely but offline in addition to pre-assembling all stimuli (audio/video/noise), we limited some of the concerns regarding exact stimulus presentation for online assessments of temporally sensitive measures like the TBW^44^. For example, in contrast to presenting the stimuli offline, hosting the experiment on a server would have led to a need to stream all the stimuli to the participants’ computer via the internet, adding uncertainty to the loading time, and hence the precise timing, for the AV stimuli. Nonetheless, even for stimulus presentations via a server, Lab.js has shown to have very low inter-trial variability (< 9 ms), for auditory, visual, and AV stimuli^45^. We acknowledge that hardware differences between the participants could result in an inflation of the intersubject test-retest reliability.

### Stimulus types

The description of the different stimulus types is limited as this was not the topic of interest for the current manuscript.

Three different levels of linguistic complexity were established (i.e., words, nonsense words, and time-reversed words and nonsense words) and used at both timepoints. At the most basic level, to establish AV correspondence without lexical information, we chose to play words or nonsense words backwards (Type C stimuli). These stimuli preserve the general temporal envelope structure, with onset and offset, amplitude changes, and phase correspondence in both modalities. In contrast to Type A or B stimuli, they break the American English language phoneme-viseme connections^46^.

The second set of stimuli (Type B) were nonsense words (e.g., kinit). These nonsense words have the same amount of phoneme-viseme information available and the same duration as words. However, unlike actual words, nonsense words lack lexical meaning. Specifically, while adhering to the syllabic structures of the language, nonsense words do not convey any lexical or semantic content. As a result, factors such as word frequency and density are not applicable to nonsense words. Finally, our third linguistic level consisted of English words (Type A).

### Temporal synchrony measure

To measure the Temporal Binding Window (TBW) a one-interval two-alternative-forced-choice simultaneity judgment task (1I-2 AFC-SJT) was employed. We asked the participant to judge whether the auditory and visual stimulus were presented at the same or different times. This simultaneity judgment task was chosen as it has been shown as the more stable measure among TBW tasks^34,35^. The video was presented simultaneously with (i.e., 0 ms SOA) or preceding the audio by various intervals (see below). Only visual-leading asynchronies were introduced as (1) auditory-leading stimuli do not occur in naturalistic speech environments^18^, (2) auditory-leading stimuli are not expected to correlate with AV speech perception performance^22,29^, and (3) to keep data collection within a timeframe that is reasonable to complete within one session.

Each trial consisted of the presentation of a fixation cross (500 ms), followed by AV stimulus presentation (4000 ms) and participant response (without time limit). The participant had to press [s] or [d] on the keyboard indicating whether the audio and video were presented at the same or different times.

Eight SOAs were selected, ranging from 0 to 500 ms asynchrony (0 ms, 50 ms, 100 ms, 150 ms, 200 ms, 300 ms, 400 ms, 500 ms), as this range encompasses the most informational part of the synchrony-asynchrony window of perception for speech stimuli^22^. All AV stimuli were generated offline using ffmpeg software (Python 3.7) to ensure the intended temporal relationship between the auditory file and its corresponding silent video file. The audio and video of all speech stimuli were recorded, and thus no dubbing or artificial changes other than introducing asynchrony by delaying the audio presentation occurred.

### Stimuli & noise fragments

The target stimuli for this experiment (i.e., Type A, B, C) were sourced stimuli that have been validated in previous work^47–52^. For each stimulus type, three different female talkers were selected, with a total of 20 different stimuli per stimulus type. Each of the eight SOAs was presented 20 times (once for every stimulus), for each of the three stimulus types, resulting in a total of 480 trials per test session.

The 480 trials per task were broken down into six blocks. The stimuli were blocked by stimulus type (3 blocks), and these were randomly broken in half (80 trials per block). The different SOA and stimulus items were intermixed within each block. All six blocks were presented in a random order. As a result, three sigmoid curves (one per stimulus type) per participant were estimated via generalized linear models with maximum likelihood estimation. The stimulus and block order was different in the two sessions for each participant.

All stimuli were presented in a four-talker-babble masker. This specific babble was used as a masker in Van Engen and colleagues^53^. Each masker fragment was a random snippet of 4000 ms, consisting of 4 female American-English talkers producing meaningful sentences (from^54^). The 4-talker-babble was equalized for RMS amplitude with Audacity 2.4.2. The speech stimulus started playing randomly 500 to 1000 ms after the start of the babble masker, with a target-to-masker ratio (TMR) of −6 dB. All target and masker files were combined in advance and offline.

All materials were processed in a similar way. iMovie was used to make all talkers similarly visible in a cut-out circle of a black frame showing head and shoulders filling 90% of height of the frame (1280 × 720 px). The length of all original videos was cut to 2000 ms, and before and after the video black frames were added to make all videos exactly 3000 ms long. Audio was split from the video fragment and Audacity 2.4.2 was used to perform amplitude equalization (dB RMS) for both stimuli and masker. Ffmpeg software (Python 3.7) was then used to select random masker fragments of 4000 ms, assemble target and masker with a TMR of −6 dB, and combine audio and video (with the selected SOAs).

### Data analysis

Statistical analyses were performed using the glm, mgcv, lme4, nlme, stats, and psych packages in R^55^ and RStudio (version 1.3.1093).

We analyzed participant-specific patterns in the relationship between temporal asynchrony and binary judgment responses (synchronous or asynchronous) at both test times. Psychometric functions were calculated based on a general linear model with a binomial distribution (logit link function), using the glm package to model the SOA-response relationship at various linguistic levels and test moments, estimating the following critical parameters: TBW width, slope and asymptote amplitude. The slope of the regression line was extracted from the model coefficients. To compute additional metrics, predictions were made at specific SOA values (between 0 ms and 500 ms in 1 ms increments), and the difference in probabilities at 500 ms and 0 ms (i.e., asymptote amplitude) was calculated. Further, we estimated the SOA value corresponding to a 50% response probability by finding the SOA value that minimized the absolute difference between predicted probabilities and 0.5.

#### Group level: TBW-shape

To analyze test-retest reliability on a group level we identified three linear mixed effects models. We used TBW width (Model 1a), slope (Model 1b), and asymptote amplitude (Model 1c) as dependent variables whereas the right half of the model (independent variables) stayed consistent: ~ stimulus type * test moment + (1 + stimulus type + test moment | participant). We used test moment 1 and Type A stimuli as references in our treatment coding, and for the random effects we included participants as random intercept, and stimulus type and test moment as random slopes. We extracted both summary and F-statistics.

#### Individual level: intrasubject variability of TBW width

To assess individual test-retest reliability we used linear correlation coefficients to test the correlation between % perceived asynchrony at test moment 1 and 2. For each stimulus type we compared scores (at each SOA) between test moments per participant. Furthermore we correlated the width of the TBW of test moment 1 and 2, per individual, split up by stimulus type. We further used linear correlation coefficients to interpret intrasubject variability of the TBW width between stimulus types.

#### Individual level: intersubject variability of TBW width

To quantify the intersubject variability we interpreted the interquartile range (IQR: Q3 - Q1) for each stimulus type. In order to measure whether the intersubject variability was significantly larger than the intrasubject variability between the two test moments, we extracted the variance components from the Model 1a, while including test moment in the random effects to interpret intercepts for each participant, and within each participant for the different test moments. We then used a likelihood ratio test to determine whether intrasubject variability (between test moments) was significantly smaller than intersubject variability.

#### Response times

The response time was analyzed between the two test moments to see whether overall response time changed by replicating the task. Given the non-normality of the residuals for the response time values we used a non-parametric generalized additive mixed model (Model 2: Response time ~ stimulus type * test moment + (1 + stimulus type + test moment | participant)) to investigate how stimulus type, test moment, and potentially the interaction between these two predicted response time, using the gamm model from the mgcv package. We implemented participants as random intercept, and stimulus type and test moment as random slopes. We used stimulus type ‘Type C’ and test moment 1 as references (treatment coding). Type C stimuli were chosen as we expected the largest effect of repeated exposure for these least familiar stimuli.

## Electronic supplementary material

Below is the link to the electronic supplementary material.

Supplementary Material 1

## Data Availability

The data that support the findings of this study are available from the first author (lgijbels@uw.edu) upon reasonable request and with the permission of the University of Washington’s review board.
