# Supplementary material for: Synchrony perception of audiovisual speech is a reliable, yet individual construct
Source: Sci Rep. 2025 May 7;15:15909. doi: 10.1038/s41598-025-00243-8 (PMC12059004; doi:10.1038/s41598-025-00243-8)
Supplement: Supplementary file 1 — Supplementary Material 1 [file 41598_2025_243_MOESM1_ESM.docx]

## Supplemental materials: Outliers

Three steps were used to define outliers, and a participant was identified as an outlier if they failed on all three steps (Table TS1).

1. Residuals were analyzed based on a generalized linear mixed effects model with binomial distribution (Model_outliers_: Percentage perceived synchronous ~ test moment * stimulus type + SOA + (1|participant)). The percentage synchrony perception was calculated for each participant, stimulus type, test moment and SOA, based on their (a)synchronous judgments. This resulted in 48 scores (3x2x8) per participant. Participant was included in the model as a random effect. We used a QQplot to analyze the residuals.
2. Scores (% perceived synchronous) were analyzed per participant (and stimulus type) for test moment 1 and 2 to identify which participant had scores more than 2 standard deviations away from the mean score of the group.
3. Response times were analyzed per participant to identify which participant had response times more than 2 standard deviations away from the mean response time. This was calculated per test moment and stimulus type.

A participant was identified as an outlier if they failed on all 3 steps.

| **Step** | **Measure** | **Criterium** | **Outlier(s)** |
| --- | --- | --- | --- |
| **1** | QQplot | Significant deviation from diagonal | P2 , P16 |
| **2** | Percentage Perceived Synchronous | > 2 standard deviations | P13 , P16 |
| **3** | Response Time | > 2 standard deviations | P2 , P4 , P10 , P16 |

###### Table TS1: Outlier measures, with criteria to classify as outlier, and the participant number of corresponding outliers.


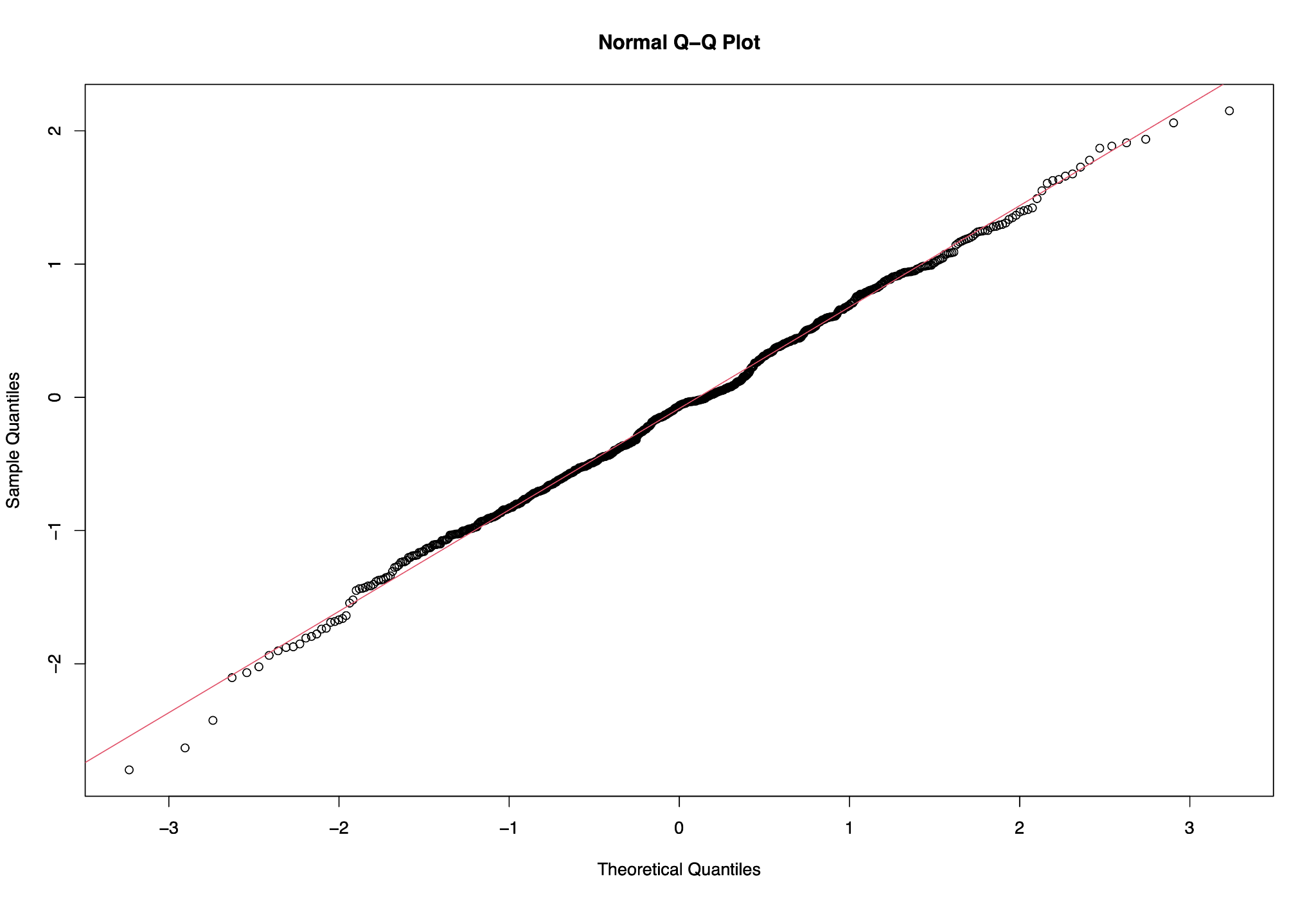


###### Figure S1: QQplot of the residuals of the Model_outliers._

Overall, the QQplot of the residual scores of the model showed a good fit for our data (Figure S1). From an analysis on an individual level we found that 2 participants (P2 and P16) acted as outliers. Based on the z-scores of the percentage perceived synchronous, averaged across SOAs but split up by stimulus type, and test moment, 2 participants (P16 and P13) had at least one value more than 2 standard deviations from the mean score. And based on the z-scores of the response time, per stimulus type and test moment, 4 participants (P16, P2, P4, P10) had at least one value more than 2 standard deviations slower than the mean score. The mean response time per trial (across participants, test moments, and stimulus types) was 3055 ms (Median = 2808 ms, sd = 781.18). P16 failed all outlier criteria, and therefore their data was excluded for all further analyses.

## Supplemental materials: Details of TBW differences at the 2 timepoints

| **Stimulus type** | **Mean Difference (ms)** | **-/+ 1 SD (ms)** | **-/+ 2 SD (ms)** |
| --- | --- | --- | --- |
| **A** | 8.04 | [-24.26 ; 40.34] | [-85.06 ; 101.14] |
| **B** | -4.92 | [-39.94 ; 30.1] | [-105.86 ; 96.02] |
| **C** | -16.0 | [-59.79 ; 27.79] | [-142.22 ; 110.22] |
| **Overall** | -4.28 | [-41.49 ; 32.94] | [-111.54 ; 102.99] |

###### Table TS2: Details of differences between TBW at T1 and T2,presenting the values in ms of the mean difference, 1 and 2 standard deviations from this mean difference; per stimulus type and across stimulus types (overall)

*
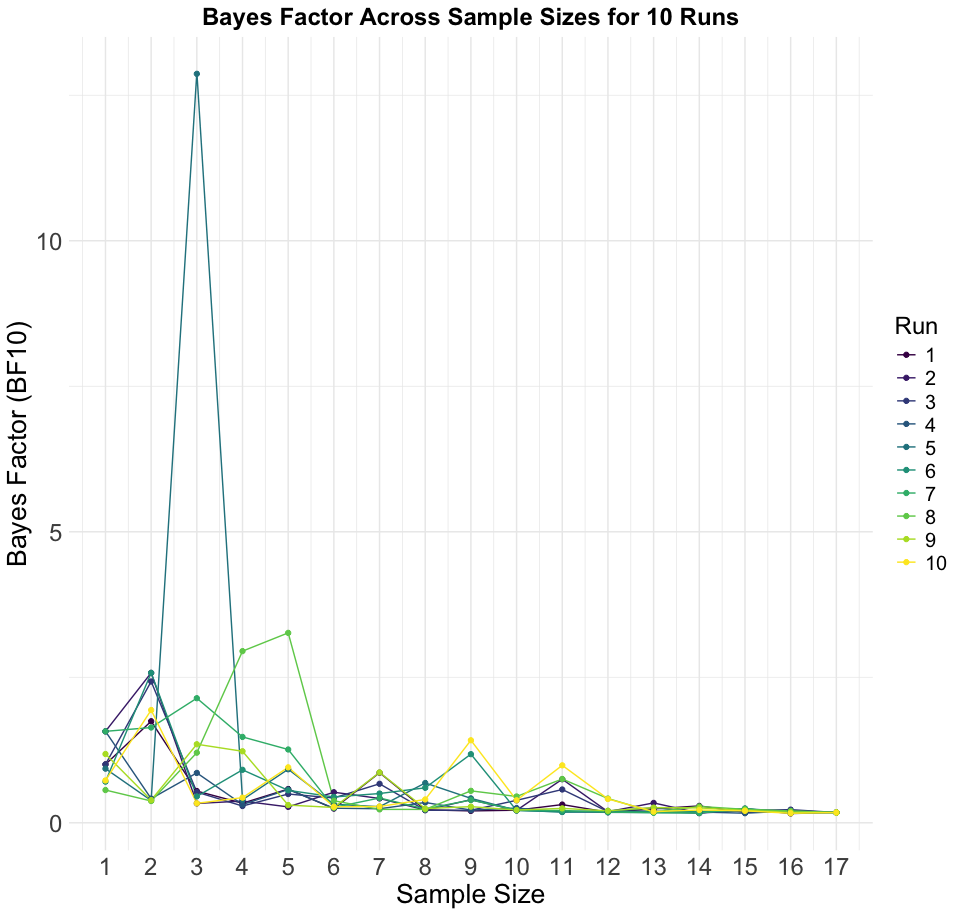
*

*Figure S2: The Bayes Factor was calculated to quantify the evidence in favor of the null hypothesis (Bayes Factor < 1). Our results (N = 17) yielded a moderate effect, a Bayes Factor of 0.18 ± 0.07%. The Bayes Factor was calculated for different sample sizes (by randomly selecting participants (N: 1 – 17) from our data) and repeated this 10 times. Stable performance shows after N > 13, suggesting a moderate sample size (N = 17) is sufficient.*
